# MALDI-TOF lipidomics rapidly detects modification of 2-hydroxymyristate lipid A, a potential virulence trait in *Enterobacter bugandensis*

**DOI:** 10.1128/spectrum.01702-24

**Published:** 2025-05-15

**Authors:** Rémy A. Bonnin, Aymeric Jacquemin, Jade Pizzato, Delphine Girlich, Cecile Emeraud, Markus Kostrzewa, Thierry Naas, Gerald Larrouy-Maumus, Laurent Dortet

**Affiliations:** 1 Team "RESIST", Université Paris-Saclay, Inserm, CEA, Center for Immunology of Viral, Auto-immune, Hematological and Bacterial diseases (IMVA-HB/IDMIT/UMRS1184) 89691 , Fontenay-aux-Roses & Le Kremlin-Bicêtre, France; 2 Bacteriology-Hygiene Unit, Bicêtre Hospital Assistance Publique-Hôpitaux de Paris, Le Kremlin-Bicêtre, France; 3 Associated French National Reference Center for Antibiotic Resistance: Carbapenemase-Producing Enterobacteriaceae, Le Kremlin-Bicêtre, France; 4 MRC Centre for Molecular Bacteriology and Infection, Department of Life Sciences, Faculty of Natural Sciences, Imperial College London 4615, London, United Kingdom; 5 Bruker Daltonik GmbH &coKG 39117 , Bremen, Germany; University of Pretoria, Pretoria, Gauteng, South Africa

**Keywords:** detection test, carbapenemase, Enterobacterales, *Galleria mellonella*

## Abstract

*Enterobacter cloacae* complex (ECC) comprises numerous species that can be difficult to identify. Among these species, *Enterobacter bugandensis* is recognized as a threat in neonatal intensive care units due to its involvement in septic shock. A potential virulence factor responsible for its virulence is the modification of the lipid A of the lipopolysaccharide (LPS). This modification corresponds to a hydroxylation of the myristate component of lipid A. A collection of 168 ECC, including 10 *E. bugandensis* and one isolate involved in fatal septic shock in the neonatal intensive care unit, were tested for the presence of 2-hydroxymyristate. All isolates were sequenced to determine the genetic diversity of this collection and detect the presence of the *lpxO* gene responsible for this hydroxylation. matrix assisted laser desorption ionization-time of flight mass spectrometry (MALDI-TOF) analysis revealed that only a fraction (4/11) of *E. bugandensis* were able to modify their lipid A, which correlates with the presence of the *lpxO* gene. Genomic analysis of *E. bugandensis* revealed that only half of the genomes carried the *lpxO* gene. Lpxo-negative isolates exhibited a deletion of 1,094 bp encompassing the *lpxO* gene. Lipid A analysis also revealed that hydroxylation of myristate was not specific to *E. bugandensis* since this modification was systematically found in several species including *Enterobacter chengduensis*, *Enterobacter chuandaensis,* or *Enterobacter cloacae subsp cloacae.* Here, we provide a simple detection test allowing the rapid identification of hydroxymyristate of lipid A involved in the virulence of *E. bugandensis*. Rapid identification of lipid A modification might help clinicians to adapt procedures when an *Enterobacter* spp. is identified in newborns.

**IMPORTANCE:**

*Enterobacter bugandensis* is now recognized as a potential threat in neonatal wards due to its ability to survive in incubators and its virulence properties. Its increased virulence is linked to the addition of 2-hydroxymyristate on lipid A, resulting from the action of the LpxO enzyme. Here, we proposed a rapid and simple assay to decipher the presence of 2-hydroxymyristate on lipid A directly from the bacterial colony. This assay can be performed easily on MALDI-TOF routine machine using a commercial kit (MBT Lipid Xtract Kit, Bruker Daltonic). This test can accurately detect the 2-hydroxymyristate on lipid A of lpxO-positive ECC isolates, including *E. bugandensis*. In neonate wards, newborns are now screened for ECC carriage. Accordingly, the MALDIxin test might help clinicians detect the presence of lpxO-positive *E. bugandensis,* which can be considered a risk factor for the newborn, and implement measures to avoid potentially fatal septicemia.

## INTRODUCTION

The genus *Enterobacter*, consisting of gram-negative rods, belongs to the order Enterobacterales and the family *Enterobacteriaceae*. The *List of Prokaryotic Names with Standing in Nomenclature* (LPSN) website inventoried 49 validly published species, including synonyms, among which 25 possessed a validated name with taxonomic status (https://lpsn.dsmz.de/, 6 December 2024). *Enterobacter* spp. are responsible for a variety of nosocomial infections including urinary tract or bloodstream infections (1).

*Enterobacter* spp. is a member of ESKAPE, encompassing seven species or genra involved in nosocomial infections associated with antimicrobial resistance (2). This genus possesses an inducible cephalosporinase (*bla*_ACT_-like gene) that can be overexpressed, leading to resistance to third-generation cephalosporins (e.g., ceftazidime and cefotaxime). Resistance to carbapenems can be due to (i) the overexpression of this AmpC associated with a decrease in membrane permeability or (ii) the production of a carbapenemase, an enzyme possessing significant hydrolysis activity toward carbapenems (3).

Nowadays, classification of *Enterobacter* species remains a complex problem. Although the heterogeneity of *E. cloacae* complex (ECC) was known and recognized, classical methodologies were not able to robustly discriminate the different species included in this complex. Thus, to overcome this problem, molecular-based methods were advocated (4). First, a classification based on partial sequencing of heat shock protein gene *hsp60* was developed to discriminate species among ECC (4). From this study, 12 clusters (I–XII) were determined. The advent of high-throughput sequencing led to a more precise classification based on phylogenetic analysis (5). This classification led to the identification of 22 clades within ECC (6).

Among ECC, Hoffmann’s cluster IX and Chavda’s clade R now corresponds to *Enterobacter bugandensis*. This species was initially identified in Tanzania during an outbreak of septicemia in a neonatal ward (7). Analysis of the genome of *E. bugandensis* EB247 revealed that this species might possess several potential virulence factors that could explain its increased virulence (8). Among these potential virulence factors, increased resistance to human serum was observed compared with non-*bugandensis Enterobacter* isolates (8). Recently, septic shocks involving *E. bugandensis* with preterm newborns were reported during an outbreak in France (9). In this study, six fatal septic shocks due to ECC were observed during a 14-month period. Whole genome analysis revealed that all these fatal septic shocks were caused by *E. bugandensis*. Interestingly, septic shocks were caused by different sequence types (STs), including ST1084 (*n* = 3), ST917 (*n* = 1), ST1085 (*n* = 1), and ST1090 (*n* = 1), strongly suggesting a particular virulence of this species rather than a clone with increased virulence (9). Recently, the role of newborn incubators as the reservoir of *Enterobacter* spp. was confirmed during another outbreak in neonatology ward (10). This study also concluded that incubators can play a role in the spread of *Enterobacter* spp., notably the species *E. bugandensis* and *Enterobacter xiangfangensis*.

A modification of lipid A, a hydroxylation of a myristate residue of lipid A, was argued to be the main weapon responsible for the increased virulence of *E. bugandensis* (11). This modification is due to the presence of the *lpxO* gene encoding an enzyme involved in 2-hydroxylation of lipid A residue. LpxO enzymes have been reported in many gram-negative rods such as, but not limited to, *Pseudomonas aeruginosa*, *Burkholderia pseudomallei,* or *Klebsiella pneumoniae* (12–14).

The aim of this study was to provide a simple diagnostic test to assess the presence of 2-hydroxymyristate on lipid A in ECC (particularly *E. bugandensis*).

## MATERIALS AND METHODS

### Strain collection

A collection of 168 *Enterobacter* spp. recovered from the “French National Reference Center for carbapenem resistance in Enterobacterales” were used in this study except for the isolate *E. bugandensis* P2B. This previously reported isolate was recovered from a fatal sepsis shock in neonatal (9). Overall, this collection of ECC comprised 16 different species including *Enterobacter asburiae* (*n* = 18), *E. bugandensis* (*n* = 11), *E. chengduensis* (*n* = 7), *Enterobacter chuanduensis* (*n* = 1), *E. cloacae subspecies cloacae* (*n* = 13), *E. cloacae subspecies dissolvens* (*n* = 1), *E. hormaechei subspecies hoffmannii* (*n* = 25), *E. hormaechei subspecies hormaechei* (*n* = 9), *E. hormaechei subspecies oharae* (*n* = 8), *E. hormaechei subspecies steigerwaltii* (*n* = 16), *E. hormaechei subspecies xiangfangensis* (*n* = 19), *Enterobacter kobei* (*n* = 6), *Enterobacter ludwigii* (*n* = 4), *Enterobacter mori* (*n* = 2), *Enterobacter quasihormaechei* (*n* = 5), and *Enterobacter roggenkampii* (*n* = 23) (Table 1).

**TABLE 1:** Peak analyses obtained on the collection of clinical isolates of *Enterobacter* spp.

Strain	Species	ST-Oxford	Peak^*a*^ intensity								% of hydroxymyristate	GenBank nucleotide accession number
			1,797 m/z	1,813 m/z	1,825 m/z	1,843 m/z	2,036 m/z	2,052 m/z	2,064 m/z	2,080 m/z		
214 E1	*E. asburiae*	358	4,617	0	6,191	0	9,991	0	11,768	0	0	SAMN47220087
268 E7	*E. asburiae*	250	9,396	0	4,840	0	8,827	0	4,174	0	0	SAMN47220127
276 E10	*E. asburiae*	657	1,072	0	485	0	1,266	0	460	0	0	SAMN47220134
151 C2	*E. asburiae*	250	262	0	368	0	860	0	1,189	0	0	SAMN47220041
166D1	*E. asburiae*	657	7,026	0	2,521	0	7,934	0	2,700	0	0	SAMN47220051
170 C2	*E. asburiae*	250	4,838	0	3,523	0	5,593	0	3,946	0	0	SAMN47220053
183D3	*E. asburiae*	250	704	0	885	0	1,850	0	1,726	0	0	SAMN47220061
186 H9	*E. asburiae*	250	909	0	511	0	1390	0	676	0	0	SAMN47220064
220D7	*E. asburiae*	250	5,375	0	6,133	0	10,092	0	12,448	0	0	SAMN47220092
247 E2	*E. asburiae*	807	1,121	0	769	0	1,227	0	380	0	0	SAMN47220108
269 G8	*E. asburiae*	484	4,848	0	3,751	0	7,382	0	2,369	0	0	SAMN47220129
172D2	*E. asburiae*	25	7,620	489	4,037	945	6,972	378	2,580	0	6.2	SAMN47220055
276I5	*E. asburiae*	53	1,285	3,225	969	2,062	8,769	3,789	1,107	1,917	22.9	SAMN47220135
282 G7	*E. asburiae*	250	2,577	0	2,061	0	3,173	0	2,113	279	0	SAMN47220142
282I7	*E. asburiae*	53	3,005	754	2,418	533	8,883	1,477	2,756	975	6.2	SAMN47220143
287I5	*E. asburiae*	252	2,203	3,689	2,298	3,337	6,124	2,855	1,538	1,810	29.5	SAMN47220151
288 F8	*E. asburiae*	24	5,983	2,410	7,114	4,828	4,671	1,131	3,054	1,335	23.7	SAMN47220153
288 G10	*E. asburiae*	252	711	1,036	911	998	5,487	1,633	1,328	1,618	14.8	SAMN47220154
P2B	*E. bugandensis*	1084	1,528	982	1,874	1,566	896	620	1,050	1,037	26.7	JADBQQ000000000
253 J3	*E. bugandensis*	901	3,515	0	5,129	0	6,400	0	7,373	0	0	SAMN47220114
118 C5	*E. nematophilus*	new	55,57	0	6,205	0	8,230	0	10,347	0	0	SAMN47220030
221 G5	*E. bugandensis*	1677	3,247	0	7,744	0	2,958	0	6,609	0	0	SAMN47220094
280D5	*E. bugandensis*	1095	1,697	0	1,824	0	6,702	0	3,609	0	0	SAMN47220138
288I5	*E. bugandensis*	499	11,887	0	9,388	0	12,907	0	9,133	0	0	SAMN47220155
291 F6	*E. bugandensis*	1677	3,035	0	4,122	0	7,412	0	5,690	0	0	SAMN47220163
296B2	*E. bugandensis*	2704	3,044	0	6,738	0	4,661	0	8,364	0	0	SAMN47220170
243 E5	*E. bugandensis*	1843	6,464	1,748	17,604	5,172	6,738	1,389	14,929	4,801	11.8	SAMN47220106
257D9	*E. bugandensis*	2069	3,520	2,644	4,699	5,396	4,171	1,988	3,402	3,635	27.3	SAMN47220115
305 C9	*E. bugandensis*	1092	6,549	6,449	12,611	18,105	7,190	6,626	11,416	16,339	28,8	SAMN47220185
204 E1	*E. chengduensis*	1065	0	2,185	1,524	12,260	1,035	2,648	1,997	16,152	38.2	SAMN47220078
215D6	*E. chengduensis*	1065	170	1,081	936	3,704	6,087	2,330	1,505	4,846	23.2	SAMN47220088
223 A3	*E. chengduensis*	1065	351	1,529	908	4,124	423	695	337	1,450	57.6	SAMN47220097
250D1	*E. chengduensis*	1065	3,856	4,400	5,953	9,108	3,398	2,881	4,031	5,511	34.5	SAMN47220111
272I4	*E. chengduensis*	598	99	617	618	2,426	2,800	858	668	2,846	27.8	SAMN47220131
283 F7	*E. chengduensis*	598	894	899	2,745	3,922	1,542	1,258	3,240	4,086	25.9	SAMN47220144
291 H5	*E. chengduensis*	598	554	1,310	2,300	5,106	3,706	1,226	1,716	4,226	31.9	SAMN47220164
228 H2	*E. chuandaensis*	1189	18,404	1,679	2,085	1,189	6,718	0	0	0	9.5	SAMN47220100
198 E8	*E. cloacae cloacae*	1540	1,531	0	7,065	3,681	960	0	4,453	1,943	18.7	SAMN47220073
199 E8	*E. cloacae cloacae*	84	3,999	783	10,122	1,885	3,570	682	8,443	1,975	8.5	SAMN47220074
166B3	*E. cloacae cloacae*	456	12,941	2,447	12,561	4,906	6,019	159	6,320	1,480	15.7	SAMN47220050
178 C6	*E. cloacae cloacae*	820	2,618	782	7,189	1,598	1,512	382	3,973	882	12.6	SAMN47220060
203 G5	*E. cloacae cloacae*	1540	4,362	1,741	11,618	7,229	2,378	790	6,857	3,490	23.3	SAMN47220077
208 C9	*E. cloacae cloacae*	412	2,947	1,093	9,055	3,265	2,451	779	7,358	2,910	14.6	SAMN47220083
229 G3	*E. cloacae cloacae*	1516	6,022	1,098	6,859	2,114	3,904	741	4,298	1,626	12	SAMN47220101
252 G4	*E. cloacae cloacae*	456	14,195	5,324	13,261	8,339	8,329	2,708	7,334	4,163	21.5	SAMN47220113
257 F5	*E. cloacae cloacae*	1718	6,626	1,757	9,467	5,229	4,457	795	5,529	2,400	19.3	SAMN47220116
267I4	*E. cloacae cloacae*	1737	85,36	2,579	4,769	1,558	6,183	1,743	2,852	867	14.2	SAMN47220125
268D8	*E. cloacae cloacae*	1718	6,686	1,622	7,361	2,422	3,725	775	4,196	1,515	14,3	SAMN47220126
290 C4	*E. cloacae cloacae*	524	8,345	534	7,078	660	6,255	274	4,850	406	4.2	SAMN47220161
300 A7	*E. cloacae cloacae*	1517	7,165	1,308	11,001	2,492	6,769	1,185	9,178	2,371	9.2	SAMN47220176
284 A3	*E. cloacae dissolvens*	1715	6,968	1,488	4,620	853	5,442	1,267	3,686	678	9.4	SAMN47220147
281 H8	*E. hormaechei hoffmannii*	764	12,269	0	7,061	0	8,160	0	2,368	0	0	SAMN47220140
296I6	*E. hormaechei hoffmannii*	102	6,119	0	2,559	0	3,435	0	1,055	0	0	SAMN47220172
300 J4	*E. hormaechei hoffmannii*	419	3,659	0	2,180	0	3,220	0	1,313	0	0	SAMN47220177
302 C10	*E. hormaechei hoffmannii*	419	4,700	0	4,398	0	2,525	0	1,931	0	0	SAMN47220179
307 G4	*E. hormaechei hoffmannii*	168	12,400	0	8,160	0	12,611	0	5,988	0	0	SAMN47220192
305 J5	*E. hormaechei hoffmannii*	310	9,933	0	7,238	0	3,539	0	2,016	0	0	SAMN47220188
128 F4	*E. hormaechei hoffmannii*	78	161,15	0	10,139	0	8,703	0	3,927	0	0	SAMN47220033
146 F7	*E. hormaechei hoffmannii*	729	14,313	0	14,285	0	7,302	0	5,963	0	0	SAMN47220037
146 F8	*E. hormaechei hoffmannii*	104	8,785	0	7,844	0	3,901	0	3,177	0	0	SAMN47220038
146 J2	*E. hormaechei hoffmannii*	419	10,950	0	6,413	0	5,338	0	2,249	0	0	SAMN47220039
155 G4	*E. hormaechei hoffmannii*	419	10,626	0	6,733	0	4,213	0	2,040	0	0	SAMN47220044
157B10	*E. hormaechei hoffmannii*	118	8,484	0	6,218	0	2,484	0	1,323	0	0	SAMN47220045
185I6	*E. hormaechei hoffmannii*	933	7,653	0	8,317	0	4,851	0	4,727	0	0	SAMN47220063
189 C3	*E. hormaechei hoffmannii*	1123	8,071	0	7,508	0	4,555	0	3,210	0	0	SAMN47220069
280 C5	*E. hormaechei hoffmannii*	1283	11,909	0	6,180	0	7,119	0	2,310	0	0	SAMN47220137
279 E3	*E. hormaechei hoffmannii*	233	13,009	1,005	10,635	0	3,822	0	1,628	0	3.3	SAMN47220136
146 A2	*E. hormaechei hoffmannii*	145	7,699	719	9,785	0	1,252	0	1,268	0	3.5	SAMN47220036
281 H4	*E. hormaechei hoffmannii*	616	13,936	1,078	10,331	0	14,170	0	5,005	0	2.4	SAMN47220139
282 C8	*E. hormaechei hoffmannii*	233	9,327	1,123	15,609	0	10,034	0	4,579	0	2.8	SAMN47220141
298 J7	*E. hormaechei hoffmannii*	1740	5,900	732	6,441	0	4,914	0	2,062	0	3.7	SAMN47220175
301 C7	*E. hormaechei hoffmannii*	683	8,586	869	15,762	0	2,674	0	2,827	0	2.8	SAMN47220178
302 G1	*E. hormaechei hoffmannii*	168	14,575	1,146	10,236	0	6,255	0	3,444	0	3.2	SAMN47220180
304I5	*E. hormaechei hoffmannii*	816	9,187	774	13,346	0	8,385	0	8,367	0	1.9	SAMN47220182
305I10	*E. hormaechei hoffmannii*	683	7,953	594	12,437	0	2,276	0	2,757	0	2.3	SAMN47220187
307 F9	*E. hormaechei hoffmannii*	97	6,416	411	6,365	0	4,588	0	3,272	0	2	SAMN47220191
251 J8	*E. hormaechei hormaechei*	528	7,632	0	9,793	0	4,339	0	4,039	0	0	SAMN47220112
259I3	*E. hormaechei hormaechei*	528	9,011	0	9,534	0	6,999	0	4,778	0	0	SAMN47220118
265 G1	*E. hormaechei hormaechei*	269	15,477	0	10,450	0	6,686	0	3,604	0	0	SAMN47220121
267 A4	*E. hormaechei hormaechei*		6,427	0	6,304	0	3,870	0	2,530	0	0	SAMN47220124
288 E6	*E. hormaechei hormaechei*	269	13,469	0	12,414	0	4,687	0	3,419	0	0	SAMN47220152
292B9	*E. hormaechei hormaechei*		10,328	0	11,430	0	13,745	0	11,754	0	0	SAMN47220165
304I10	*E. hormaechei hormaechei*	528	3,293	0	8,161	0	1,757	0	4,075	0	0	SAMN47220183
172I10	*E. hormaechei hormaechei*	528	7,763	553	12,245	0	4,593	0	6,038	0	1.8	SAMN47220057
309 A6	*E. hormaechei hormaechei*	269	11,525	1,319	26,212	0	6,621	0	12,891	1,566	2.2	SAMN47220194
188D3	*E. hormaechei oharae*	108	6,200	863	32,224	0	3,135	0	15,841	0	1.5	SAMN47220068
220 H7	*E. hormaechei oharae*	108	2,540	434	9,098	0	1,962	0	5,371	0	2.2	SAMN47220093
152 E1	*E. hormaechei oharae*	1068	6,215	706	17,811	0	2,099	0	6,218	0	2.1	SAMN47220042
170I2	*E. hormaechei oharae*	108	2,227	0	8,463	0	685	0	2,672	0	0	SAMN47220054
269 A7	*E. hormaechei oharae*	108	6,011	0	11,631	0	2,338	0	4,500	0	0	SAMN47220128
295I10	*E. hormaechei oharae*	1086	4,654	0	20,443	0	10,786	0	17,174	0	0	SAMN47220168
297 A1	*E. hormaechei oharae*	108	1,838	0	9,459	0	743	0	3,848	0	0	SAMN47220173
310 C1	*E. hormaechei oharae*	108	1,814	0	7,474	0	607	0	2,874	0	0	SAMN47220195
306D9	*E. hormaechei steigerwaltii*	1116	20,442	0	40,992	0	9,384	0	18,378	0	0	SAMN47220189
248 E7	*E. hormaechei steigerwaltii*	113	4,755	0	2,276	0	2,279	0	1,038	0	0	SAMN47220109
222 J2	*E. hormaechei steigerwaltii*	831	4,302	0	8,144	0	3,292	0	4,809	0	0	SAMN47220096
223 C7	*E. hormaechei steigerwaltii*	51	3,120	0	6,260	0	903	0	1,580	0	0	SAMN47220098
224 C9	*E. hormaechei steigerwaltii*	90	6,354	0	10,724	0	4,255	0	5,566	0	0	SAMN47220099
237 C9	*E. hormaechei steigerwaltii*	45	7,913	0	6,354	0	3,017	0	1,738	0	0	SAMN47220103
285B3	*E. hormaechei steigerwaltii*	111	27,827	0	32,949	0	18,371	0	15,431	0	0	SAMN47220148
285 F6	*E. hormaechei steigerwaltii*	346	2,089	0	1,240	0	2,388	0	790	0	0	SAMN47220149
285 H5	*E. hormaechei steigerwaltii*	177	19,409	0	12,105	0	9,023	0	4,066	0	0	SAMN47220150
289 F10	*E. hormaechei steigerwaltii*	742	40,859	0	32,530	0	18,721	0	10,313	0	0	SAMN47220158
290B6	*E. hormaechei steigerwaltii*	106	17,183	0	659	0	9,972	0	0	0	0	SAMN47220159
290I4	*E. hormaechei steigerwaltii*	175	11,090	1,212	19,746	0	3,506	0	3,725	0	3.1	SAMN47220162
295 J1	*E. hormaechei steigerwaltii*	1296	7,930	742	17,455	0	3,796	0	6,713	0	2	SAMN47220169
283 J10	*E. hormaechei steigerwaltii*	110	14,524	1,852	23,342	0	4,504	0	6,501	0	3.7	SAMN47220146
283 J8	*E. hormaechei steigerwaltii*	50	3,400	724	5,976	0	1,351	0	1,339	0	5.7	SAMN47220145
296B7	*E. hormaechei steigerwaltii*	106	5,851	4,493	7,006	6,872	5,233	3,190	4,067	3,864	28	SAMN47220171
137I4	*E. hormaechei xiangfangensis*	114	3,359	0	9,591	0	1,185	0	3,984	0	0	SAMN47220034
139I8	*E. hormaechei xiangfangensis*	66	499	0	1,693	0	0	0	743	0	0	SAMN47220035
149I6	*E. hormaechei xiangfangensis*	182	7,792	0	19,685	0	1,802	0	8,479	0	0	SAMN47220040
176B5	*E. hormaechei xiangfangensis*	109	1,037	0	7,284	0	335	0	1,919	0	0	SAMN47220059
184D7	*E. hormaechei xiangfangensis*	1261	1,101	0	7,214	0	2,188	0	3,616	0	0	SAMN47220062
192 F1	*E. hormaechei xiangfangensis*	109	10,296	0	31,172	0	3,141	0	9,997	0	0	SAMN47220070
192 H5	*E. hormaechei xiangfangensis*	143	1,527	0	5,768	0	1,458	0	3,358	0	0	SAMN47220072
201 F6	*E. hormaechei xiangfangensis*	171	772	0	5,693	0	1,540	0	2,378	0	0	SAMN47220076
208 H2	*E. hormaechei xiangfangensis*	459	1,669	0	8,410	0	988	0	5,095	0	0	SAMN47220084
220 A10	*E. hormaechei xiangfangensis*	148	2,571	0	20,576	0	4,506	0	9,246	0	0	SAMN47220091
222B6	*E. hormaechei xiangfangensis*	1077	8,935	0	21,219	0	4,521	0	9,299	0	0	SAMN47220095
157 E2	*E. hormaechei xiangfangensis*	98	119,502	0	9,245	0	33,495	0	0	0	0	SAMN47220046
205I8	*E. hormaechei xiangfangensis*	303	10,844	1,351	44,091	0	3,152	0	9,693	0	2	SAMN47220079
205 J10	*E. hormaechei xiangfangensis*	356	5,062	781	24,251	0	936	0	4,671	0	2.2	SAMN47220080
211 E4	*E. hormaechei xiangfangensis*	121	8,621	1,124	35,253	0	5,563	0	20,263	0	1.6	SAMN47220085
213 E10	*E. hormaechei xiangfangensis*	136	0	0	197	0	0	0	0	440	0	SAMN47220086
175 A9	*E. hormaechei xiangfangensis*	171	8,540	8,922	19,553	23,818	4,900	3,943	9,529	11,128	36.2	SAMN47220058
187 C4	*E. hormaechei xiangfangensis*	527	2,932	0	12,391	8,412	2,664	0	9,457	7,055	19.6	SAMN47220066
199I1	*E. hormaechei xiangfangensis*	245	9,922	10,516	36,887	49,106	4,052	4,950	15,114	22,525	39	SAMN47220075
270 C3	*E. kobei*	32	9,575	3,245	11,187	3,478	8,902	2,935	10,538	4,277	12.4	SAMN47220130
162 E3	*E. kobei*	125	2,710	2,028	2,186	2,443	1,838	1,378	1,653	1,904	27.7	SAMN47220049
243 E3	*E. kobei*	56	1,668	0	1,906	760	3,245	1,051	2,851	1,490	5.9	SAMN47220105
161 E4	*E. kobei*	87	7,757	2,006	9,949	5,266	4,307	1,021	6,104	2,636	18.6	SAMN47220048
260 J7	*E. kobei*	910	1,918	0	2,445	0	3,985	0	2,849	0	0	SAMN47220119
261B10	*E. kobei*	1034	249	0	157	0	504	0	273	0	0	SAMN47220120
238 H9	*E. ludwigii*	13	1,767	0	0	0	1,170	0	0	0	0	SAMN47220104
265 H2	*E. ludwigii*	374	684	0	0	0	345	0	0	0	0	SAMN47220122
265 H3	*E. ludwigii*	374	18,513	0	2,334	0	11,167	0	1,051	0	0	SAMN47220123
293 H7	*E. ludwigii*	20	7,915	0	0	0	6,713	0	0	0	0	SAMN47220166
62 F4	*E. mori*	130	10,950	0	0	0	6,250	0	0	0	0	SAMN47220029
120 J4	*E. mori*	1006	3,882	0	0	0	3,491	0	0	0	0	SAMN47220032
118 G6	*E. quasihormaechei*	873	3,001	0	5,011	0	4,414	0	5,298	0	0	SAMN47220031
153 C2	*E. quasihormaechei*	487	1,170	0	2,954	0	2,605	0	3,298	0	0	SAMN47220043
207 H2	*E. quasihormaechei*	873	5,608	0	8,067	0	6,578	0	8,210	0	0	SAMN47220082
245I7	*E. quasihormaechei*	873	4,413	0	5,709	0	6,345	0	5,493	0	0	SAMN47220107
289 A7	*E. quasihormaechei*	873	3,000	0	3,409	0	3,627	0	3,013	0	0	SAMN47220156
172D4	*E. roggenkampii*	165	3,059	0	2,581	0	0	0	0	0	0	SAMN47220056
186 J1	*E. roggenkampii*	595	1,779	0	1,617	0	1,588	0	1,419	0	0	SAMN47220065
206 C2	*E. roggenkampii*	96	408	0	1,108	0	648	0	1,548	0	0	SAMN47220081
217 F8	*E. roggenkampii*	997	1,446	0	0	0	0	0	0	0	0	SAMN47220089
258 A9	*E. roggenkampii*	561	1,085	0	612	0	637	0	475	0	0	SAMN47220117
275 C8	*E. roggenkampii*	561	1,647	0	1,250	0	946	0	831	0	0	SAMN47220132
276 A9	*E. roggenkampii*	1614	3,102	0	2,482	0	1,700	0	1,579	0	0	SAMN47220133
308B3	*E. roggenkampii*	165	1,267	0	1,622	0	1,295	0	1,804	0	0	SAMN47220193
219 E6	*E. roggenkampii*	166	579	0	692	0	799	0	965	0	0	SAMN47220090
159 A4	*E. roggenkampii*	1576	1,150	0	2,153	1,536	674	0	1,501	981	19.2	SAMN47220047
168 A8	*E. roggenkampii*	595	1,124	443	0	0	358	117	0	0	21.7	SAMN47220052
187 G7	*E. roggenkampii*	515	296	0	924	320	937	330	2,176	1,207	5.2	SAMN47220067
192 H1	*E. roggenkampii*	523	3,807	0	2,938	0	6,155	401	4,428	0	0	SAMN47220071
234D7	*E. roggenkampii*	1594	3,476	702	3,913	731	2,293	386	2,669	504	9.8	SAMN47220102
249D5	*E. roggenkampii*	1259	1,605	263	2,021	332	1,087	0	1,306	0	9	SAMN47220110
289 F4	*E. roggenkampii*	1614	1,549	212	1,549	269	935	119	894	0	8.7	SAMN47220157
290B7	*E. roggenkampii*	486	1,833	0	0	0	1,954	0	570	203	0	SAMN47220160
293 J9	*E. roggenkampii*	826	1,777	0	2,307	0	2,796	0	3,699	2,539	0	SAMN47220167
298I3	*E. roggenkampii*	595	556	0	886	0	1,301	0	2,102	1,985	0	SAMN47220174
303 C5	*E. roggenkampii*	515	99	0	413	374	287	174	628	690	14	SAMN47220181
304 J9	*E. roggenkampii*	595	4,311	694	7,268	2,930	2,620	0	5,052	1,859	14.7	SAMN47220184
305 G5	*E. roggenkampii*	165	2,391	266	3,136	602	0	0	0	0	13.6	SAMN47220186
307 F3	*E. roggenkampii*	1134	1,569	0	2,764	1,090	1,444	0	2,819	835	10.4	SAMN47220190

^*a*^ Peaks corresponding to 2-hydroxymyristate modified lipid A are highlighted in gray.

### DNA extraction and whole genome sequencing

Genomic DNA of *Enterobacter* isolates were extracted using the GeneJet genomic DNA extraction kit according manufacturer’s recommendations (Thermo Fischer Scientific, Les Ulis, France). Whole genome sequencing based on Illumina’s technology was performed as previously described (15). After sequencing, raw data were assembled *de novo* using the CLC genomics v.21 program (Qiagen, Les Ulis, France), and the genomes were analyzed online using software applications available at the Center for Genomic Epidemiology-CGE (https://www.genomicepidemiology.org/).

### Bioinformatic analysis of *E. bugandensis* lpxO genes

In addition to our collection of *E. bugandensis*, all available sequences of *E. bugandensis* available in Genbank on the 1st March 2024 were included. A sequence search was performed with the alignment-search tool BLAST+ with all *E. bugandensis* whole genome sequences as subjects and a 909 bp long sequence of *lpxO* originated from *E. bugandensis*. The *lpxO* gene was searched by BLAST analysis using 80% nucleotide identity over 90% coverage of the gene as criteria. The phylogenetic analysis of the data set consisted of a phylogenetic tree inferred by the maximum likelihood algorithm IQ-tree (best-fit model automatically selected by ModelFinder). It was based on an alignment of the core genome of the data set. The alignment was done using MAFFT, and the core genome was constructed using Panaroo with default parameters.

### MALDIxin-based detection of 2-hydroxymiristate modification of lipid A

A volume of 150 µL bacterial suspension was pipetted into a 1.5 mL Eppendorf tube, centrifuged at 15,000 × *g* for 5 min, and then washed twice with 400 µL double distilled water (ddH_2_O). Lipid A from *Enteobacter* strains was extracted using the MBT Lipid Xtract Kit (Bruker Daltonic, Bremem, Germany), following the manufacturer’s instructions. Briefly, 100 µL of bacterial suspension was suspended in 50 µL of the MBT Lipid Xtract hydrolysis buffer. Then, 44 µL of the cell suspension was discarded, and the remaining 6 µL was submitted with the lid of the tube closed to a heating process at 90°C for 30 min. The tubes were left for 2 min with the lid open to completely evaporate the buffer. The dried pellets were washed with 50 µL of the MBT Lipid Xtract washing buffer for a few seconds without dissolving the pellet. The total volume of the washing buffer was discarded by pipetting. Finally, 5 µL of the matrix was pipetted up and down for 15–20 s to resuspend the dried pellet, and 2 µL of the suspension was spotted onto either an MSP 96 polished steel target (Bruker Daltonics, Part-No. 8280800) or a MBT Biotarget 96 (Bruker Daltonics, Part-No. 1840375).

To calibrate the MALDI, 0.5 µL peptide calibration standard II (Bruker Daltonik) together with 0.5 µL of its corresponding calibration matrix were loaded onto the target plate along with the samples to be detected. A mixture of 0.4 µL water and 1.2 µL matrix was used as a negative control. Spectra were acquired on MALDI Biotyper Syrius automated using the linear negative-ion mode (laser intensity: 65%, laser frequency: 200.0 Hz, voltage: 15 kV, pulsed ion extraction: 200 ns, detection range: 820–4200 *m/z*). FlexAnalysis software (Bruker Daltonik) was used for baseline subtraction and peak detection of the spectra.

### *Galleria mellonella* larva infection model

Wax moth larvae (INRA UMR-1319, Université Paris Saclay, Jouy en Josas, France) were used on the day of reception. Larvae with a cream-colored cuticle presenting minimal speckling or discoloration, weighing 200–300 mg and measuring 2–2.5 cm in length were used for each experiment. Four isolates of *E. bugandensis* were used for worm virulence assays. Two *lpxO*-positive isolates (P2B and 305C9) and two *lpxO*-negative isolates (253J3 and 291F6). Overnight bacterial cultures in LB were diluted 1:10 and grown to an OD_600_ of 0.4–0.6. Cultures were centrifuged and bacterial cells were resuspended in physiological sterile water and serially diluted from 10^9^ CFU/mL to 10 CFU/mL. An inoculum of 10^5^ CFU was injected (10 µL of 10^7^ CFU/mL) into the hindmost proleg of healthy larvae (3 × 10 animals per group) using a microinjector (Hamilton syringe model 802 RNW 25 µL, Fisher Scientific, Illkirch, France). Physiological water (10 µL) was injected in the control group. Then, injected larvae were incubated at 37°C for 96 h. Larvae were considered dead when they displayed no movement in response to touch and had turned black. The survival Kaplan-Meier curves were drawn using the GraphPad Prism software (version 6.0 c). Statistical analysis (log-rank test) was performed on three independent experiments (3 × 10 animals per condition) using GraphPad Prism v10 software.

## RESULTS

### ECC collection analysis

A collection of 168 clinical isolates of representative members of the *Enterobacter* genus were used in this study. Sequencing followed by genetic analysis revealed a wide genetic diversity. Overall, a total of 118 different STs were identified over the 168 isolates. Of note, 10 different STs were identified among the 11 *E. bugandensis* isolates. A wide genetic diversity was of great importance for the tested collection to avoid a bias related to a clonal relationship. In our collection, most of the ECC produced a carbapenemase, including VIM, OXA-48-like, NDM, and IMI, reflecting the French epidemiology identified in *Enteroabcter* spp (16, 17).

### MALDIxin-based detection of 2-hydroxymiristate modification of lipid A

The analysis of lipid A of the 168 EC isolates of our collection was performed using MALDIxin protocol on a clinical microbiology routine MALDI-TOF mass spectrometer, MALDI Biotyper Syrius. Peaks analyses are presented in Table 1.

**Fig 1:**
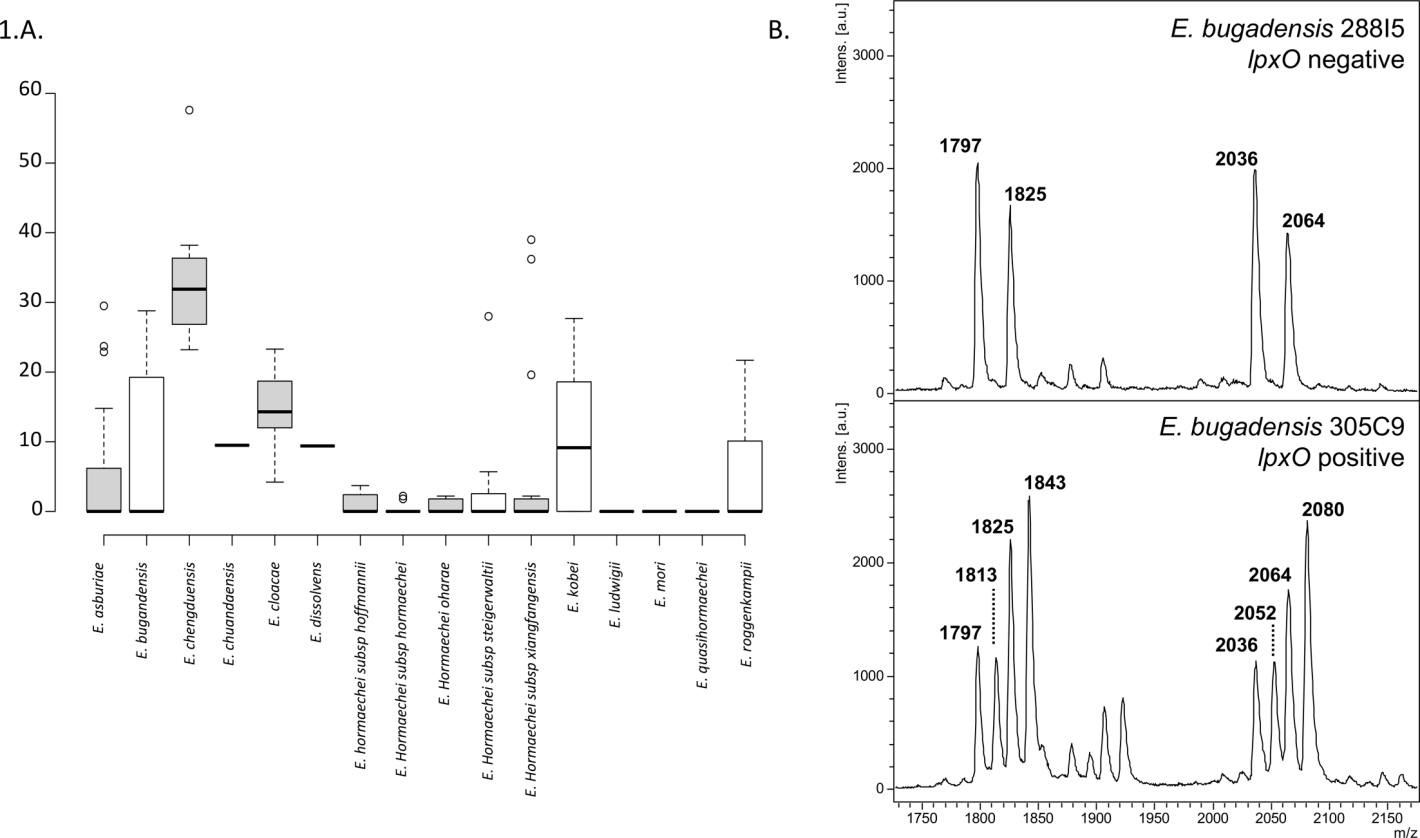
(A) Percentage of hydroxymyristate detected in the different *Enterobacter* species. Median and quartile are represented. (B) Peak analysis of two representative *E. bugandensis* isolates possessing or not *lpxO* gene. m/z values of the peak are indicated.

As shown in Fig. 1B, the mass spectrum of *E. bugandensis* 288I5 (*lpxO*-negative isolate) is dominated by a set of two peaks assigned to bis-phosphorylated hexa-acyl lipid A and bis-phosphorylated hepta-acyl lipid A. The major peak at *m/z* 1797 corresponds to hexa-acyl diphosphoryl and lipid A, respectively, containing four C14:0 3-OH, one C14:0, and one C12:0 (18). The peak at m/z 2035 corresponds to hepta-acyl diphosphoryl lipid A four C14:0 3-OH, one C14:0, one C12:0, and one C16:0. Peaks at m/z 1825 and m/z 2064 are also observed in *E. bugandensis,* which can tentatively be assigned to hexa-acyl diphosphoryl lipid A, containing four C14:0 3-OH and one C14:0, and hepta-acyl diphosphoryl lipid A, containing five C14:0 3-OH, one C14:0, and one C16:0, respectively. In *E. bugandensis* 305C9 (*lpxO*-positive strain), additional peaks were identified at *m/z* 1813, *m/z* 1843, *m/z* 2052, and *m/z* 2080 corresponding to the addition of an hydroxyl group on myristic acid on the native fatty acid chain (11) and annotated as to the 2-hydroxymyristate modified lipid A.

The ratio of 2-hydroxymyristate modified lipid A was calculated by dividing the intensities of peaks 2-hydroxymyristate modified lipid A (*m/z* 1813, *m/z* 1843, *m/z* 2052, and *m/z* 2080) by the sum of intensities of all peaks (unmodified + 2-hydroxymyristate modified peaks) (Table 1).

Ratio analysis of lipid A 2-hydroxymyristate modification revealed that (i) this lipid A modification is not unique to *E. bugandensis* and (ii) this property is not shared by all members of a species (Fig. 1A; Fig. S1). For instance, in three species *E. ludwigii, E. mori,* and *E. quasihormaechei*, no 2-hydroxymyristate branched on lipid A could be identified, whereas all representatives of *E. chengduensis* possessed this modification on their lipid A (Fig. 1A).

In most cases, only some isolates of the species were able to branch this 2-hydroxy-myristate. As an example, in *E. bugandensis*, only four of the 11 tested strains produced a 2-hydroxymyristate modified lipid A, suggesting that the locus involved in this modification did not belong to the core genome of *E. bugandensis*.

Of note, a variable level of 2-hydroxymyristate modified lipid A can also be observed alongside the *Enterobacter* species. *E. chengduensis* isolates presented a high rate of 2-hydroxymyristate modified lipid A compared with other species. In this species, all isolates (*n* = 7) presented a modified version of lipid A. However, it should be noted that the seven tested *E. chengduensis* isolates corresponded to only two different STs (ST1065 and ST598).

### Role of 2-hydroxymyristate-branched lipid A in the virulence of *E. bugandensis*

**Fig 2:**
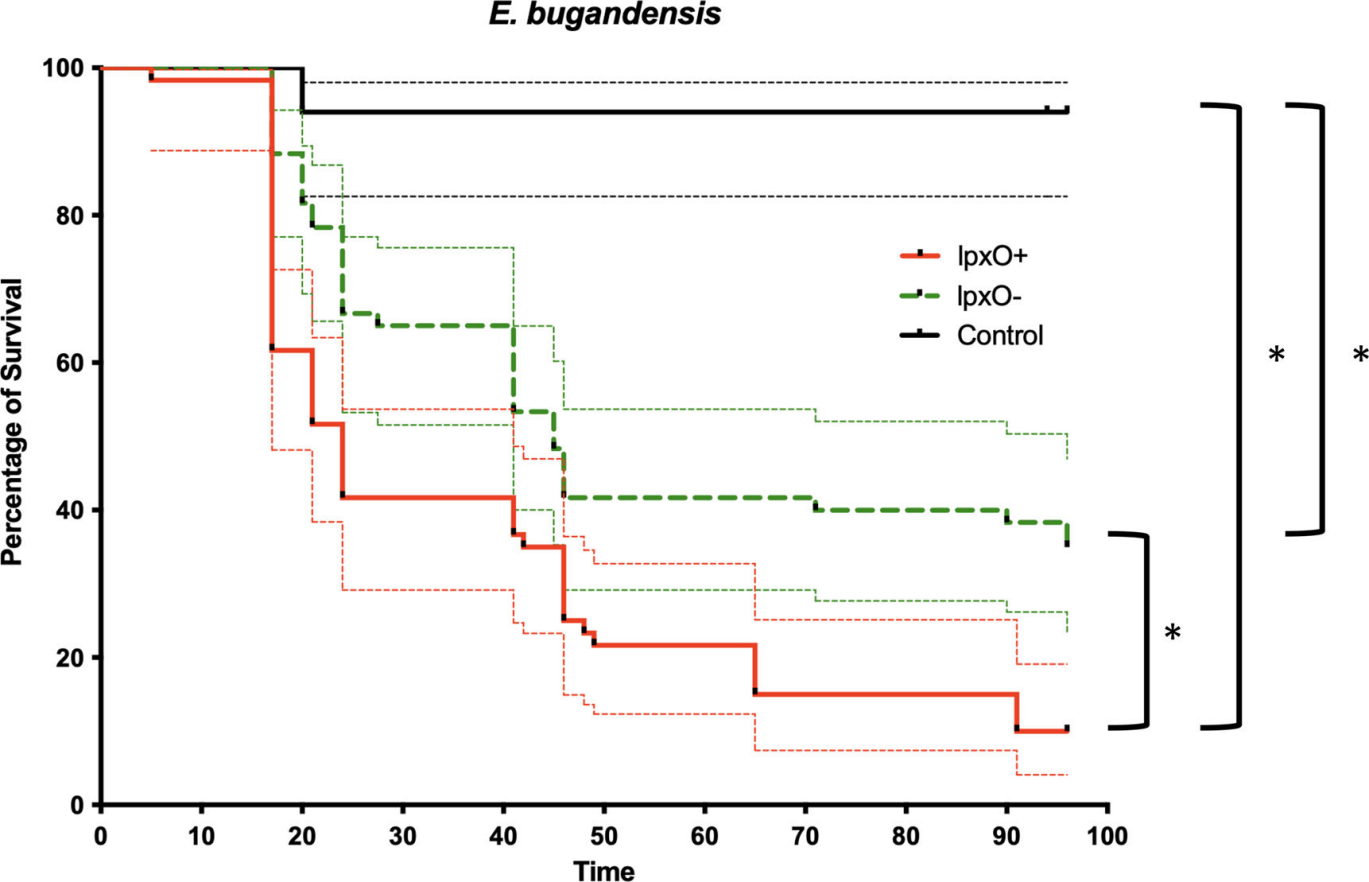
Survival rates of *G. mellonella* infected by either *lpxO*-positive or -negative isolates or by physiological water. LpxO-positive isolates used were isolates P2B & 305C9 and lpxO-negative isolates were 291F6 & 253J3. Survival rates were performed over a 4-day period and with 3 × 10 animals per group. A star indicated a *P* value < 0.001.

It has been advocated that 2-hydroxymyristate-branched lipid A is a major virulence factor in *E. bugandensis* (11). To confirm this hypothesis, *Galleria mellonella* survival assay was performed with *E. bugandensis* exhibiting lipid A with or without 2-hydroxymyristate according to MALDIxin results (Fig. 2). Four isolates were selected and tested in triplicate. Two isolates displaying 2-hydroxymyristate modified lipid A, *E. bugandensis* P2B isolated from a blood culture sample of a preterm neonate who died from a fatal septic shock (ST1084) (9) and 305C9 (ST1092), and two isolates with native lipid A, strains 253J3 (ST901) and 291F6 (ST1677). Over a time span of 4 days post-inoculation, a significant decrease in survival was observed for the two isolates displaying 2-hydroxymyristate-modified lipid A. A median of survival at 24% was observed for *lpxO*-positive *E. bugandensis* versus a median of 45% for *lpxO*-negative isolates. Log-rank Mantel-Cox test indicated that these curves were different with a *P* value < 0.001 (Fig. 2). It confirmed the involvement of 2-hydroxymyristate lipid A modification in the virulence of *E. bugandensis*.

### Distribution of lpxO in *E. bugandensis*

**Fig 3:**
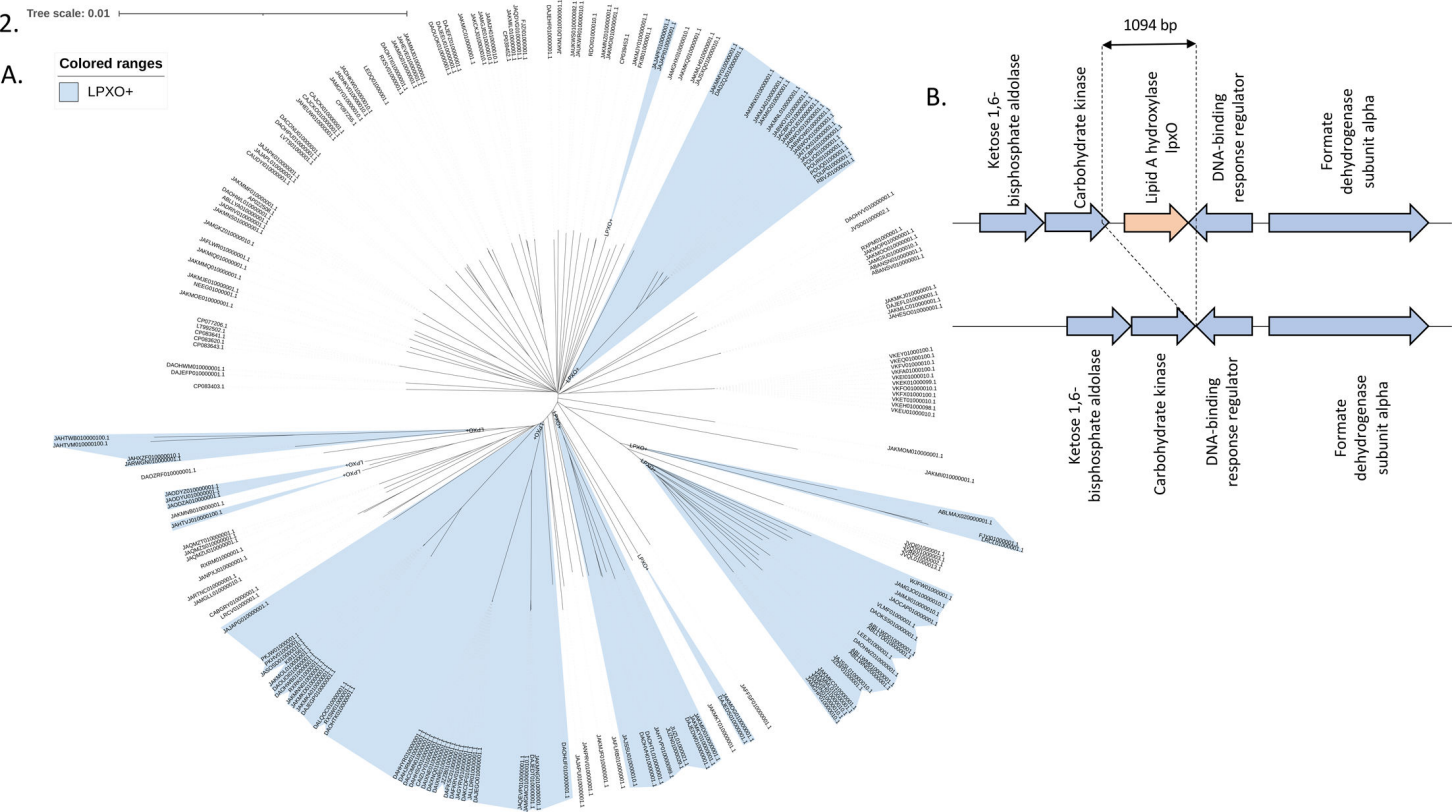
(A) Distribution of the *lpxO* gene in *E. bugandensis*. All available genomes in Genbank on the 1^st^ of March 2024 were included. The *lpxO* gene was searched by blast analysis with 80% nucleotide identity over 90% coverage of the gene. GenBank accession numbers are indicated on the tree. Isolates possessing the *lpxO* gene are indicated in blue. (B) Genetic context analysis of the *lpxO* gene. The *lpxO* gene is indicated in orange and other genes in blue.

The addition of 2-hydroxymyristate is the consequence of several successive lipid A modification involving LpxM, LpxL, and LpxO enzymes. However, the final 2-OH hydroxylation is mediated by the lipid A hydroxylase LpxO. This enzyme is encoded by the *lpxO* gene. Among the 11 *E. bugandensis* tested isolates, only four exhibited a 2-hydroxymyristate-modified lipid A according to MALDIxin results (Table 1). It suggested that either *lpxO* did not belong to the core genome of *E. bugandensis* or this gene is not expressed in all genetic backgrounds. To answer this question, a collection of 209 *E. bugandensis* genomes from our collection and from the Genbank database were tested for the presence of *lpxO* (Fig. 3A). Among these 209 genomes only 97 were positive for the presence of *lpxO*. Unexpectedly, the presence of *lpxO* was not distributed by phylogenetic branches with a common ancestor but rather distributed across several branches as usually observed for acquired mobile genetic elements. Accordingly, the close genetic environment of *lpxO* was analyzed to search for potential mobile genetic elements potentially involved in its acquisition. Intriguingly, this analysis did not reveal the presence of any mobile elements (Fig. 3B). Instead, in *lpxO*-negative isolates, a deletion of 1,094 bp systematically occurred. This deletion encompassed the whole *lpxO* gene and the 3’ extremities of the two surrounding genes without a trace of any genetic rearrangement linked to mobile element excision.

## DISCUSSION

*E. bugandensis* is now recognized as a potential threat in neonatal wards due to its ability to survive in incubators and its virulence properties (9, 10). This increased virulence was proposed to be linked to a modification of lipid A. This modification corresponded to the addition of 2-hydroxymyristate (11). This study aimed to propose a rapid and simple assay to decipher the presence of 2-hydroxymyristate on lipid A directly from the bacterial colony by using MALDI-TOF technology with MBT lipid Xtract assay (Bruker Daltonik, Germany). We tested the robustness of the test on a collection of 168 *Enterobacter* spp. clinical isolates belonging to 16 different species. This test demonstrated that (i) different species can modify their lipid A by the addition of 2-hydroxymyristate and (ii) this modification can be present in only some isolates within the same species. Indeed, *E. bugandensis* is not the sole species able to modify its lipid A by adding a 2-hydroxymyristate, and among the *E. bugandensis* tested isolates, only four over seven gave a positive result. This observation prompted us to look for the presence of the incriminated gene involved in this modification, *lpxO*. In our collection of seven *E. bugandensis* isolates, the four strains modifying their lipid A possessed a chromosome-encoded *lpxO* gene. Overall, this gene was present only in half of all *E. bugandensis* genomes present in the NCBI database (Fig. 2). Unfortunately, we could not determine the reason for the lipid A can be the absence/presence of *lpxO* in these genomes. Indeed, in *lpxO*-negative strains, we observed a systematic deletion of 1,094 bp in size in the bacterial chromosome. Since no genetic mobile elements were identified bracketing this gene or a larger portion, the gene loss in these genomes could be hypothesized but remained enigmatic.

We previously demonstrated that *E. bugandensis* exhibited a higher virulence compared with other *Enterobacter* species in a *G. mellonella* model (9), and this result was confirmed using a mouse model in which LPS from *E. bugandensis* demonstrated higher immunogenic properties (11). Here, we tested four clinical isolates of *E. bugandensis* (two with and two without the *lpxO* gene) in a *G. mellonella* model. We observed a clear difference between these two conditions confirming the role of *lpxO* in the virulence of *E. bugandensis*. However, an interesting point should be highlighted; despite this lipid A modification being involved in virulence, its role might not be sufficient to explain the global virulence of *E. bugandensis* in neonates. Indeed, some other ECC species such as *E. chengduensis* or *E. xiangfangensis* also include isolates displaying a similar rate of 2-hydroxymyristate modification (Table 1; Fig. 1A) but were never reported in neonatal fatal sepsis shock. Accordingly, deeper investigations are still remaining to fully understand the particular virulence of *E. bugandensis* in neonates. Nevertheless, the presence of *lpxO* leading to the final 2-hydroxymyristate modification of lipid A can be considered an additional risk factor if *E. bugandensis* is involved.

Since the MALDIxin assay can be performed easily on the routine machine using a ready-to-use commercial kit (MBT Lipid Xtract Kit, Bruker Daltonic), this test could be performed directly on *E. cloacae* complex colonies cultured from a screening sample (such as rectal swabs) or from clinical samples recovered from a neonate or from during an environmental sampling of incubators in the neonatal ward.

In the reported fatal cases of septic shocks in newborns, *E. bugandensis* isolates were susceptible to β-lactams, and thus, treatment was microbiologically efficient but infection led to early septic shock responsible for the patient death (9). This tragic issue resulted in modifications to patient monitoring. Indeed, in the neonate ward, newborns are now screened for ECC carriage. Accordingly, the MALDIxin test might help clinicians detect the presence of *lpxO*-positive ECC (particularly if *E. bugandensis* is suspected) and implement measures to avoid potentially fatal septicemia. We assume that this test possesses some limits since it is not specific to *E. bugandensis*. However, an ECC positive for the presence of 2-hydroxymyristate on lipid A can be considered a risk factor for the newborn. The procedure following the identification of 2-hydromyristate-lipid A ECC remains to be determined. They can include decolonization procedures based on the result of the antibiogram, increased surveillance of the newborn, or implementation of hygiene measures regarding invasive procedures or catheter manipulations.

This MALDIxin assay can also be useful to test ECC strains cultured from environmental sampling of incubators. Indeed, incubators were identified as environmental sources of ECC-related outbreaks in neonatal intensive care units (9, 10). The identification of ECC isolates with 2-hydroxymyristate-modified lipid A could indicate the presence of a *lpxO*-positive *E. bugandensis* that can be considered a risk factor for newborns. Accordingly, supplemental hygiene measures such as incubator disinfection or incubator replacement might be considered to limit the risk of further infection of preterm neonates.

To conclude, we provided a simple and rapid test to decipher the presence of 2-hydroxymyristate on lipid A of ECC isolates. This method can decipher the presence of modified lipid A rather than just the presence of *lpxO* gene that might not be expressed. Although this modification of lipid A is not only present in *E. bugandensis*, *lpxO*-positive *E. bugandensis* is now recognized as a potential threat of sepsis in the neonatal ward. Thus, the MALDIxin assay could be helpful to identify the presence of this pathogen and implement control measures. In addition, this assay used the MALDI-TOF machine already available in many clinical microbiology laboratories for the routine identification of bacterial colonies. Of note, despite being commercially available, the MBT Lipid Xtract Kit required a routine MALDI-TOF machine able to work in a negative mode, which is not the case for the machines of all manufacturers. Indeed, bacterial identification only required the usage of the positive mode (detection of positively charged proteins), whereas the MBT Lipid Xtract Kit allows the identification of negatively charged lipids including lipid A.

## SUPPLEMENTAL MATERIAL

Figure S1 spectrum.01702-24-s0001.pdf Percentage of Hydroxymiristate detected in the n=11 *E. bugandensis*. Isolates possessing the lpxO gene are indicated.

ASM does not own the copyrights to Supplemental Material that may be linked to, or accessed through, an article. The authors have granted ASM a non-exclusive, world-wide license to publish the Supplemental Material files. Please contact the corresponding author directly for reuse.
