# Supplementary material for: MALDI-TOF lipidomics rapidly detects modification of 2-hydroxymyristate lipid A, a potential virulence trait in Enterobacter bugandensis
Source: Microbiol Spectr. 2025 May 15;13(6):e01702-24. doi: 10.1128/spectrum.01702-24 (PMC12131795; doi:10.1128/spectrum.01702-24)

Figure S1. Percentage of Hydroxymiristate detected in the 11 *E. bugandensis*. Isolates possessing the *lpxO* gene are indicated.

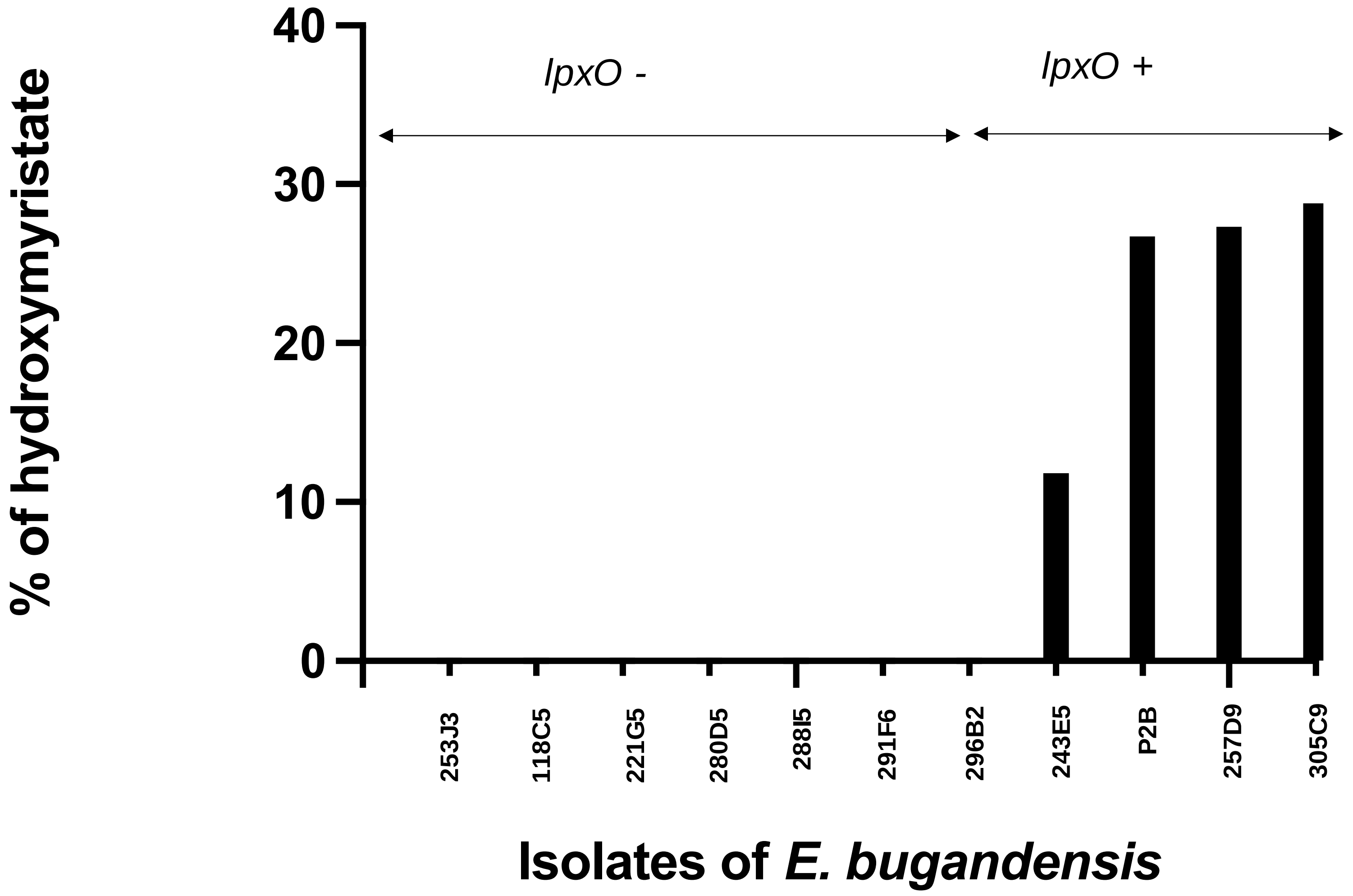

Supplement: Figure S1 — Percentage of Hydroxymiristate detected in the n=11 E. bugandensis. Isolates possessing the lpxO gene are indicated. [file spectrum.01702-24-s0001.pdf]
